# Psychometric properties of long‐term care needs questionnaire for older Chinese adults with dementia

**DOI:** 10.1111/jjns.12367

**Published:** 2020-08-11

**Authors:** Ning Sun, Rangcheng Jia, Chunyan Guo, Tongda Sun, Xiaoxin Dong, Long Li

**Affiliations:** ^1^ NingBo College of Health Sciences Ningbo China; ^2^ Ningbo City College of Vocation Technology Ningbo Ningbo China

**Keywords:** evaluation, long‐term care needs, older adults with dementia, psychometrics

## Abstract

**Aim:**

Long‐term care needs are important for older adults with dementia in the West, but they have not received enough attention from China. This study evaluated the psychometric properties of a long‐term care needs instrument for older Chinese adults with dementia.

**Methods:**

A total of 1,212 older adults with dementia were recruited from five Chinese cities to complete a 30‐item long‐term care needs questionnaire. The reliability and validity of the questionnaire was evaluated using multiple assessments, including a content validity assessment, Cronbach's alpha, an item‐to‐total correlation test, and exploratory factor analysis.

**Results:**

The questionnaire was divided into four sub‐sections: life care services (eight items), basic and specialist care services (12 items), mental comfort psychological services (four items), and homecare support services (six items). Cronbach's alpha was .93 for the whole questionnaire and ranged from .83 to .92 for the four sub‐questionnaires. The item‐to‐total correlation coefficients for the four sub‐questionnaires were between .68 and .88, and the test–retest correlation coefficient was .88.

**Conclusion:**

Our results validated the reliability and validity of a questionnaire designed to measure the quality of long‐term care services for older Chinese adults with dementia.

## 1 INTRODUCTION

Senile dementia is a progressive neurodegenerative disease the main clinical manifestations of which include progressive memory impairment, general mental decline, personality changes, and mental behavior abnormalities (Alzheimer's Disease International, 2015). According to the 2015 China Retirement Agency Development Research Report, there are an estimated 10 million dementia sufferers in China. This cohort accounts for 25% of the world's total number of adults with dementia (Yaozhong, 2016). China's population is aging, and consequently the number of older adults with dementia is increasing. In 2018, the number of Chinese people requiring long‐term care was estimated to be between 60 million and 70 million, affecting more than 300 million people (Yaozhong, 2016).

A standardized long‐term needs assessment tool thus needs to be developed in order to optimize limited old‐age service resources and meet the service needs of dementia sufferers. Long‐term care is defined as providing a series of health care, personal care, and social services to people who are incapacitated or have not had a certain degree of mobility for a period of time (Gu, 2019). Assessment tools will be of great significance for the standardization and scientific management of dementia care services, the realization of the goal of a healthy aging society, the formulation of a national mental health pension policy, and the provision of suitable care services for older adults with dementia. A literature search determined that nine elements cited with high frequency were found to be related to the demand for long‐term care services: activities of daily living score, mental health status, spouse status, children's situation, family economic level, awareness of long‐term care services, service professional, fee level, and government support (Gu, 2019; Mosca, van der Wees, Mot, Wammes, & Jeurissen, 2017).

Internationally, service providers in the long‐term care system for dementia sufferers are generally composed of interdisciplinary team members, but the composition of their care services varies due to different service models and social systems. In the US PACE service model, long‐term care services include doctors, nurses, social workers, dietitians, occupational therapists, physiotherapists, and entertainment therapists (Crump, Repin, & Sutherland, 2015), and long‐term care in this model includes home care, community settlement facilities, and institutional care (Yinan, 2011). Family care is further divided into health care and life care. The Canadian Long‐Term Home Care Program offers three types of services: rehabilitation care, family life care, and end‐of‐life care (Mosca et al., 2017). Home care in the UK can be divided into personal care, such as washing, dressing, eating, and other daily care; household assistance, such as cleaning, shopping, and making beds; and medical services, such as dressing and medication. Depending on the amount of services required, the UK model can be further divided into high‐intensity home care (≥10 hr and ≥6 visits per week), moderate‐intensity home care (5–10 hr per week and ≤6 visits), and low‐intensity family nursing (≤5 hr per week) (Ward et al., 2018). In general, the higher the level of care service, the longer the time required to deliver the service and the more complex its content. Basic home care is provided by nurses and family caregivers living with the person with dementia; nurses or health workers visit the home to help with the medical care based on the instructions of the attending doctor; physical therapists or occupational therapists visit to help with functional training; and surgeons, dentists, pharmacists, and other physicians may also be called on to assist (Hongyan, Li, Jialin, et al., 2013).

At present, there is no clear standard for grading care in China's old‐age dementia care services industry. In China, families, institutions, and communities provide the main long‐term model for taking care of elderly dementia sufferers (Wang & Tsay, 2012; Youhua, 2012; Zhengcheng, 2013). Although they have corresponding service specifications, the standard of care is not uniform. Community home care services mainly include life care, medical care, and spiritual comfort. There are also scholars in China (Kan & Wei, 2009) who argue that long‐term care services for the disabled elderly should be further refined to include personal care, health care, psychosocial services, residential services, general care services, and hospice care. Some community‐based service providers have established a service standard that includes food care, sanitation services, agency services, accompanying medical services, social interaction, and other daily care services (Hui, 2012). For example, Beijing, Tianjin, and other places have established home care service centers in the community. In addition to providing daily services such as day care and meals to the elderly, they also provide services such as home care and rehabilitation care for disabled elderly people with limited mobility or special needs (Fang, 2013).

The following characteristics hold for current domestic and foreign studies. (a) The sample of most studies was normal ordinary older people. There were very few studies of the long‐term care needs of older people with dementia. (b) The service contents largely concerned those for ordinary older people than for older adults with dementia. (c) The measuring tools were mostly constructed based on the researcher's own experience, and an evaluation tool for the long‐term care needs of older people with dementia has not yet been developed. Accordingly, this study evaluates the psychometric properties of a long‐term care needs instrument designed for older Chinese adults with dementia. The construction of a long‐term care needs instrument for older people with dementia is imperative to help the government achieve evaluation standardization and scientifically improve management levels.

## 2 METHODS

### 2.1 Study design

This quantitative study was conducted using a psychometric properties questionnaire.

### 2.2 Setting and sample

A total of 1,500 older adults with dementia were identified and selected for this study by convenience sampling from five Chinese cities: Ningbo, Qingdao, Chengdu, Shijiazhuang, and Shanghai. The sample from each city comprised 300 older adults with dementia selected using convenience sampling. All older adults with dementia were diagnosed in the clinic.

The following inclusion criteria were used: (a) from local households and older than 60 years of age; (b) met the Diagnostic Criteria for Dementia (4th edition of the *American Diagnostic and Statistical Manual of Mental Disorders*), and were diagnosed with *mild dementia* by relevant specialists; (c) were receiving mostly home‐based care; and (d) had the ability to finish the questionnaire by themselves. Exclusion criteria: (a) unconsciousness; (b) severe dementia; and (c) impaired communication.

Sample size calculation: older adults with dementia who were older than 60 years of age in China accounted for about 12% of the elderly population (P), and the sampling survey tolerance *d* = 0.15*P*, α = .05, *t* ≈ 2, *Q* = 1 − *P*, according to the sample survey size calculation formula *n* = $\;\frac{t_{\alpha }^{2}\mathrm{PQ}}{d^{2}}$ .The minimum necessary sample size of older adults with dementia required for this survey was calculated as 1,303. Given the likelihood of invalid questionnaires, a 15% missing rate was used for calculation. The total sample required was 1,500.

### 2.3 Data collection

Ten investigators underwent unified training. The data collection was conducted by face‐to‐face interviews. All older adults with dementia stayed in their home or nursing home. All the older adults with dementia were included in this study after a scheduled meeting arranged by nursing home and community service center managers according the list of clinical diagnoses. The investigators explained the research objectives and methods, and obtained consent and cooperation from older adults with dementia and their families who met the inclusion and exclusion criteria. The older adults with dementia who consented to participate received an envelope containing a packet with the questionnaires. Participants completed the questionnaires immediately upon receipt and replaced them in the envelope for collection by the investigators. To ensure anonymity, code numbers were placed on the completed questionnaires after their return to the investigators.

### 2.4 Study measures

The long‐term care needs questionnaire was compiled by the research team. The research team made statements based on all the correlated references and experts in related fields. The final decision regarding the wording used in the Chinese version of the instrument was made after a formal discussion with five experts in the field of care of the aged. The selection criteria of the five experts were as follows: (a) had 5 years or more experience caring for older adults with dementia; (b) had a master's degree and above; (c) and a title of deputy senior and above. The questionnaire comprised 30 statements. Each statement scored on a five‐point scale: *none*, *a little*, *some*, *many*, and *a lot* (1–5 points, respectively), where the higher the score, the higher the demand. The long‐term care needs questionnaire was confirmed to contain 30 statements according to all correlated references and formal discussion with five experts. The 30 statements were categorized into four sections according to formal discussion with five experts: life care services (eight items: housekeeping service, transportation, etc.), basic and specialist care services (12 items: regular physical examination, provision of safe medication guidance, etc.), mental comfort psychological services (four items: psychological counseling services, cultural and recreational activities, etc.), and home care support services (six items: legal advice, training for home caregivers, etc.).

### 2.5 Ethical considerations

The research was approved by the Ningbo College of Health Sciences Ethics Review Board (NBWY‐011). All the older adults with dementia were included in this study after a scheduled meeting arranged by nursing home and community service center managers in which the candidates were briefly explained that they had the right to withdraw at any time. All the dementia participants who consented or were authorized to take part received the questionnaire. All participants were asked to place their completed questionnaire in an envelope to ensure confidentiality. A code number was then placed on each of the completed questionnaires by the investigators for anonymity.

### 2.6 Data analysis

The software Statistical Package for Social Sciences (version 22.0, SPSS Inc., Chicago, IL, USA) was used to perform the statistical analyses of the data in the study. Differences were considered significant at *p* < .05. The mean imputation method developed by Polit and Beck (2004) was used to process the missing values. The questionnaire was sent to five administrators at five nursing homes and community service centers located in five Chinese cities. The selection criteria of the five experts were as follows: (a) had 5 years or more experience caring for older adults with dementia; (b) working in nursing homes or community service centers; (c) and a title of deputy senior and above. Before implementation, an expert panel was established to assess the content validity of the questionnaire following the method proposed by Lawshe (1975). Subsequently, a content validity ratio (CVR) was calculated according to the formula $\mathrm{CVR}=\frac{\mathrm{ne}-N/2}{N/2}$, where *ne* refers to the number of panelists who indicated “fairly relevant” or “very relevant” in response to a specific question in the questionnaire, and *N* represents the total number of panelists. In addition, all the administrators were required to answer a final question about the relevance of the whole questionnaire by selecting one of four possible answers: *do not need at all*, *do not need*, *need somewhat*, or *need totally*. Exploratory factor analysis was used to determine the construct validity of the questionnaire, and PCA was performed to derive an independent sub‐questionnaire out of the total 30‐item questionnaire. Varimax rotation was used for extraction. To evaluate the constructed sub‐questionnaire, four factors were chosen in the extraction of the factor analysis, and a loading level of 0.50 was used for the items to be included in each component. To test the internal consistency of the instrument, Cronbach's alpha coefficients were calculated for the full questionnaire and the four sub‐questionnaires (Polit & Beck, 2004). Test–retest reliability was assessed in 100 older adults with dementia. Test–retest reliability was equal to the intraclass correlation coefficient of the scores obtained by the same group of subjects in the two tests. The interval between the two tests was 3 weeks. An item‐total correlation analysis was performed to assess the potential correlation between items. A correlation coefficient of .40 or higher was accepted (Spector, 1992). The test–retest reliability was also assessed using 100 older adults.

## 3 RESULTS

The response rate was 80.80% (1,212/1,500). There were 206 questionnaires that had incomplete data. A total of 82 older adults with dementia who did not want to finish the questionnaire withdrew from the study. All subjects included in this study had dementia and were aged between 60 and 105 years. Their average age was 80.77 years. Their monthly income ranged from 500 to 9,000 yuan, with an average income of 3,752.49 yuan. There were 519 female (42.8%) and 693 male (57.2%) participants. Most of the older adults with dementia had a primary school education degree (*n* = 277, 22.9%), and most were widowed (*n* = 663, 54.7%).

Content validity ratios ranging from 0.8 to 1.0 were obtained from expert panelists. To determine the structure validity of the questionnaire, a factor analysis was performed, and the Kaiser–Meyer–Olkin (KMO) measure was found to be 0.97, and the Bartlett test yielded extremely significant results (*χ* ^2^ = 28,903.206; *p* < .0001). In the factor analysis, these four factors accounted for 77.8% variance of 30 items. (Table 1).

**TABLE 1:** Factor loading for long‐term care needs

Item	Eigenvalue	% explained variance	Communality	Derived varimax factor solution factors			
				1	2	3	4
**Basic and specialist care services**	19.390	64.633					
Item 18			0.858	.738	.242	.446	.235
Item 17			0.842	.719	.225	.458	.255
Item 9			0.762	.698	.318	.373	.185
Item 16			0.799	.673	.428	.189	.356
Item 10			0.765	.668	.356	.297	.323
Item 19			0.802	.658	.275	.418	.345
Item 15			0.807	.656	.437	.225	.367
Item 13			0.805	.628	.571	.111	.268
Item 12			0.784	.616	.521	.154	.331
Item 14			0.775	.587	.524	.106	.380
Item 20			0.786	.487	.364	.457	.455
Item 11			0.602	.472	.342	.044	.410
**Life care services**	1.651	5.503					
Item 3			0.776	.301	.758	.328	.055
Item 5			0.755	.408	.726	.218	.118
Item 1			0.751	.204	.684	.478	.113
Item 2			0.783	.237	.663	.367	.390
Item 4			0.806	.302	.661	.364	.380
Item 7			0.812	.200	.635	.286	.535
Item 6			0.684	.514	.624	.053	.169
Item 8			0.782	.300	.622	.291	.470
**Mental comfort psychological services**	1.317	4.391					
Item 21			0.867	.264	.250	.833	.201
Item 22			0.896	.295	.265	.813	.278
Item 23			0.858	.251	.288	.789	.299
Item 24			0.651	.134	.279	.556	.496
**Home care support service**	.968	3.226					
Item 27			0.708	.332	.077	.262	.723
Item 26			0.792	.319	.283	.445	.642
Item 25			0.760	.365	.261	.429	.612
Item 30			0.776	.294	.506	.315	.579
Item 29			0.699	.550	.120	.241	.568
Item 28			0.786	.411	.468	.380	.503

Older adults with dementia were highly perceptive of their long‐term care needs (mean = 13.50; standard deviation [SD] = 4.01). A general belief among older adults with dementia was that they had the greatest access to both basic and specialist care services (mean = 3.54, *SD* = 1.06) and life care services (mean = 3.42, *SD* = 1.04). A Cronbach's alpha coefficient of .93 was calculated for the full questionnaire, and for the four sub‐questionnaires ranged from .83 to .92 (Table 2). Correlation coefficients ranging from .68 to .88 were obtained in the item‐to‐total analysis (Table 3). Among these, the highest correlation coefficients were found for the items in the support sub‐questionnaire. All 30 items in the full questionnaire were observed to have an adjusted item‐total correlation coefficient of more than .60. The test–retest intraclass correlation coefficient was .88 for the two tests.

**TABLE 2:** Detailed description and reliability of the long‐term care needs questionnaire

The questionnaire and its sub‐questionnaire	Mean	*SD*	Score range	The number of items	Cronbach's alpha coefficients
Life care service	3.42	1.04	1–5	8	.89
Basic and specialist care services	3.54	1.06	1–5	12	.92
Mental comfort psychological service	3.20	1.17	1–5	4	.85
Home care support service	3.21	1.12	1–5	6	.83
Total questionnaire	13.50	4.01	4–20	30	.93

**TABLE 3:** Correlations between item and total long‐term care needs questionnaire

Items	Item‐to‐total correlation
Life care services	
1 housekeeping service	0.74
2 transportation	0.83
3 catering services	0.74
4 home safety environment guidance, advice and improvement	0.86
5 people to look after washing, dressing, etc.	0.76
6 excretion care	0.72
7 community day care (care during the day, home at night)	0.83
8 behavior management (daily life ability training, anti‐lost wearable equipment)	0.84
Basic and specialist care services	
9 regular physical examination	0.80
10 provision of safe medication guidance	0.84
11 guides	0.70
12 provision of oral care	0.84
13 regular rollovers, partial massage and other methods to prevent or treat pressure sores	0.82
14 guide measures to reduce pain and implement pain relief	0.83
15 provision of emergency services	0.86
16 specialist care services	0.85
17 health files	0.83
18 regular health assessments and recommendations	0.84
19 health consultation and health education	0.85
20 directives for prevention and self‐help methods in dangerous situations	0.84
Mental comfort psychological services	
21 on‐site companionship (chat, newspaper, medical treatment, etc.)	0.88
22 psychological counseling services	0.88
23 cultural and recreational activities	0.74
24 pairs of help activities	0.80
Home care support services	
25 legal advice, legal aid	0.78
26 training for home caregivers	0.71
27 home care provider's rest service	0.82
28 guide to purchase, lease and use of protective gear	0.82
29 hospice care	0.68
30 family beds	0.74

## 4 DISCUSSION

The questionnaire had good validity. CVRs ranging from 0.8 to 1.0 were obtained from expert panelists. All five experts agreed that the full questionnaire was relevant. The items loaded on the four factors were in line with each sub‐questionnaire. The high factor loadings of items on each factor accorded with Waltz et al.'s recommendation of at least 0.4 for factor loadings (Waltz, Strickland, & Lenz, 2005). It is acceptable that individual factors could explain 3.2% to 64.6% of the variance (Polit & Hungler, 1997). The explanation of 77.8% of the variance by the four factors was consistent with the recognized standard that an ideal factor analysis explains 40% to 60% of the variance (Wood & Haber, 2002). Our factor analysis results were consistent with the original four‐factor model. This evaluation further suggests that the questionnaire is valid, reliable and suitable for the use as a long‐term care needs measurement for older adults with dementia.

The questionnaire had good reliability. Reliability is a prerequisite for questionnaire design (Polit & Hungler, 1997). Cronbach's alpha is an important indicator of reliability in a questionnaire that is used to assess the internal consistency of the sub‐sections in the full questionnaire and their respective reliabilities (Salkind, 2000). The questionnaire designed here was deemed reliable and valid, and the items in the questionnaire were relevant to the long‐term care needs of older adults with dementia. In addition, an item‐to‐total correlation analysis was performed to validate the questionnaire's reliability (Wood & Haber, 2002). The high item‐total and test–retest correlation coefficients further suggest the reliability of the questionnaire.

In this study, a four‐factor model including life care services, basic and specialist care services, mental comfort psychological services, and home care support services was obtained by item analysis and factor analysis. Table 2 shows that basic and specialist care services and life care services were in demand among older Chinese adults with dementia, and these service needs were similar to those of dementia sufferers in Western nursing homes (Lin, Otsubo, & Imanaka, 2017; Tobis, Wieczorowska‐Tobis, Talarska, Pawlaczyk, & Suwalska, 2018). It was shown that caregivers should provide more basic, specialist care, and life care services for older adults with dementia in China. The results were also similar to those of corresponding dementia sufferers in China nursing homes (Gu, 2019). All the long‐term care in the Western model was supplied by home care, community settlement facilities, and institutional care (Mosca et al., 2017; Yinan, 2011). Their division of labor was different in detail. In China, long‐term care is crucial in home care. Community and institutional functions need to be further developed to improve the quality of life of older adults with dementia.

### 4.1 Limitations and further research

One limitation of the present study is that it was a convenience sample and that our researchers only included participants from five Chinese cities. The second limitation is that our study did not verify the relationships between long‐term care needs and long‐term care outcomes such as quality of life and cognitive status. A larger study is definitely needed to evaluate the utility value of the long‐term care needs questionnaire in the future.

## 5 CONCLUSION

At present, the problems associated with an aging Chinese population are increasing, especially in cities. The large number of only‐child families, the accelerated pace of modern life, and the increasing demands of the working environment have led to problems associated with the long‐term care of older adults with dementia as well as a more pressing shortage of available care. This heavy burden on caregivers may also hinder economic development. This study constructed a long‐term care service demand scale for older adults with dementia and evaluated the results of its administration to a large sample. By analyzing the reality of China's old‐age care, we found that care for older adults with dementia was in high demand, especially in the areas of access to basic and specialist care services and life care services. The study is theoretically significant for professionals working directly with older adults with dementia and those in related research fields, and has the potential to assist in building a more effective care service system for dementia sufferers. It is important to reliably evaluate and validate the potential long‐term care needs of older adults with dementia. However, further investigation should include the assessment of long‐term care outcomes such as quality of life and cognitive status outcomes, rather than just the long‐term care needs of older Chinese adults with dementia.

## 6 RELEVANCE TO CLINICAL PRACTICE

Our results validated the reliability and validity of a questionnaire designed to measure the quality of long‐term care services for older Chinese adults with dementia. By analyzing the reality of China's old‐age care, we found that care for older adults with dementia was in high demand, especially in the areas of access to basic and specialist care services and life care services. The study is significant for professionals working directly with older adults with dementia and those in related research fields, and can help the government standardize evaluation methods to scientifically improve the management level of this sector.

## CONFLICT OF INTERESTS

The authors declare they have no involvement, financial or otherwise, that may potentially bias their work.

## AUTHORS' CONTRIBUTIONS

The authors were responsible for the paper as follows: NS, conception, design, analysis, and data interpretation, drafting the manuscript, revising the manuscript, and its final approval; LL, acquisition of data, project administration, manuscript revisions, and its final approval; CYG, formal analysis, manuscript revision, and final approval; TDS and XXD, conception, manuscript revision, and final approval; and RCJ, conception, design, funding acquisition, project administration, manuscript revision, and final approval. All the authors have read and approved the final manuscript.
